# 1157. Determining the Clinical Utility of 16S rRNA in the Management of Pediatric Infections

**DOI:** 10.1093/ofid/ofab466.1350

**Published:** 2021-12-04

**Authors:** Peter Paul Lim, Ankita P Desai, Sree Sarah Cherian, Sindhoosha Malay

**Affiliations:** 1 UH Rainbow Babies and Children’s Hospital, Cleveland, Ohio; 2 University Hospitals/Rainbow Babies and Children’s Hospital, Cleveland, OH; 3 University Hospitals - Cleveland Medical Center, Cleveland, Ohio; 4 University Hospitals - Cleveland Medical Center/ Case Western Reserve University, Cleveland, Ohio

## Abstract

**Background:**

Conventional culture remains the gold standard to facilitate a targeted antimicrobial regimen in the treatment of bacterial infections. However, certain pediatric infections are caused by fastidious organisms and treatment with antibiotics prior to specimen collection may hamper growth of pathogens in routine culture. The use of 16S rRNA in culture negative infections has improved identification of bacterial pathogens in select scenarios. However, the specific impact of 16S rRNA on clinical decision making, especially in pediatric infections, is not well-defined. This study aims to elucidate the utility of 16S rRNA on clinical management of pediatric infections.

**Methods:**

A retrospective analysis was done on different clinical specimens which had 16S rRNA performed from August 2016 – March 2020 in our institution. Detailed chart review was performed to determine how the 16S rRNA result impacted clinical decision making. Clinical utility was defined as change in patient’s overall antimicrobial regimen, pathogen confirmation, and treatment duration.

**Results:**

Seventy-four samples from 71 pediatric patients were included in the analysis: 32 (43%) were fluid specimens and 42 (57%) were tissue specimens. Significant clinical utility was identified in 30 (40.5%) of 74 clinical samples (*p* < 0.0001). Of all specimens, pulmonary samples yielded the most clinical utility (n=9, 30%) followed equally by joint fluid (n=6, 20%) and bone (n=6, 20%). There was no significant difference in clinical utility between fluid and tissue specimens (*p=* 0.346). In 64 patients whose antimicrobial spectrum coverage was analyzed, patients with broad spectrum coverage was decreased from 48 to 21 and narrow spectrum coverage increased from 16 to 43 using 16S rRNA result, though not significant (*p*= 0.4111). Of all patients included in the analysis, the median number of antibiotics used before 16S rRNA result, 2, was significantly decreased to 1 (*p* < 0.0001).

**Conclusion:**

16S rRNA has a significant impact in terms of decreasing number of antibiotics used in treatment of pediatric infections. Pulmonary specimens have the highest clinical utility among all samples. Additional cost benefit analysis needs to be completed to further determine clinical benefit.

**Disclosures:**

**All Authors**: No reported disclosures

